# 967 Clinical Impact of Time to Surgery Following Burn Center Admission

**DOI:** 10.1093/jbcr/iraf019.498

**Published:** 2025-04-01

**Authors:** Eva Murphy, Anastasiya Ivanko, Jonathan Schoen, Herbert Phelan, Bart Phillips, Randy Kearns, Jeffrey Carter

**Affiliations:** Tulane University School of Medicine; Burn Center at University Medical Center; Louisiana State University Health Sciences Center - New Orleans; Louisiana State University Burn Center; BData, Inc; University of New Orleans; University Medical Center New Orleans

## Abstract

**Introduction:**

Burn injuries continue to be a significant cause of disability and suffering in the United States, despite numerous prevention programs. The availability of specialized burn care is limited, with only 135 burn centers among nearly 6,000 hospitals in the United States. Burn centers are specialized facilities with medical services and resources tailored to optimize burn care and rehabilitation. This study aims to evaluate the clinical impact of the time to surgery following burn center admission.

**Methods:**

The American Burn Association’s (ABA) Burn Care Quality Platform (BCQP) database was queried and analyzed by the burn research team in April 2024. The BCQP includes 103 participating burn centers with data elements from over 450,000 cases. Our base population was formed on initial burn admissions from 2020-2022 for both pediatric (0-17 years) and adult (18+) age groups. Patients with trauma diagnoses, as defined by the American College of Surgeons, were excluded. Days from admission to time of first burn surgery (excluding escharotomies) was defined using ICD-10 codes. We analyzed only complete data fields which included: burn etiology and total body surface area, patient gender, and length of stay.

**Results:**

The initial cohort included 70,490 subjects (n=16,631 aged 0-17, n=53,859 aged 18+). After applying exclusion criteria, the final cohort comprised 61,243 (n=14,915 aged 0-17, n=46,328 aged 18+) burn patients. Of the pediatric cases, 47.8% (7,130) had surgery, with a mean time to surgery of 2.2 days, while 52.2% (7,790) were managed non-operatively. Among adults, 54.4% (25,190) had surgery, with a mean time of 2.9 days, while 45.6% (21,140) were non-operatively managed. Both age groups showed earlier surgery trends for larger burns.

**Conclusions:**

Our study indicates a trend towards earlier surgery for larger burns in both pediatric and adult patients. The proportion of surgical to non-surgical management differs between these age groups, potentially reflecting variations in burn etiology. Early surgical intervention, beneficial for both small and large burns, could potentially improve outcomes, reduce long-term disability, and enhance recovery by minimizing complications. Early surgical intervention also offers significant cost savings by shortening hospital stays and reducing rehabilitation needs. Future research should analyze cost and reimbursement strategies to confirm the economic benefits of early surgery, optimizing resource use and improving burn care nationwide.

**Applicability of Research to Practice:**

This study’s findings are applicable to medical practice by guiding optimal timing for burn surgery. Early surgical intervention, especially in larger burns, improves outcomes, reduces complications, and shortens recovery times. It also presents opportunities for cost savings by minimizing hospital stays and rehabilitation needs. Tailoring care based on age and burn severity can further enhance the quality and efficiency of burn care.

**Funding for the Study:**

N/A

